# The Living Lab Concept in the Detection, Prevention and Monitoring of Geriatric Syndromes in Elderly Patients with Cardiovascular Disease—A Narrative Review

**DOI:** 10.3390/jcm15124745

**Published:** 2026-06-18

**Authors:** Anca-Iuliana Pîslaru, Ramona Ștefăniu, Mihaela-Cristina Panait (Baghiu), Mădălina Istrate, Sabinne-Marie Albișteanu, Bogdan-Cristian Brumă, Ana-Maria Turcu, Iulia-Daniela Lungu, Adina-Carmen Ilie, Ionuț Nistor

**Affiliations:** 1 Grigore T. Popa University of Medicine and Pharmacy, 700115 Iași, Romania; anca.morosanu2@umfiasi.ro (A.-I.P.); madalina.istrate@umfiasi.ro (M.I.); sabinne-marie.taranu@umfiasi.ro (S.-M.A.); bogdan-cristian-bruma@email.umfiasi.ro (B.-C.B.); ana-maria_turcu@umfiasi.ro (A.-M.T.); lungu.iulia-daniela@d.umfiasi.ro (I.-D.L.); adina.ilie@umfiasi.ro (A.-C.I.); ionut.nistor@umfiasi.ro (I.N.); 2 Technical University Gheorghe Asachi, 700050 Iași, Romania; cristina.baghiu@digital-innovation.zone; 3 Center for Technology Transfer—Mavis, Grigore T. Popa University of Medicine and Pharmacy, 700358 Iași, Romania

**Keywords:** living lab, geriatric syndromes, cardiovascular disease, elderly, monitoring

## Abstract

**Background:** Population ageing has increased the burden of geriatric syndromes among older adults with cardiovascular disease, where frailty is associated with adverse outcomes, including hospitalization, functional decline, and mortality. Digital technologies and Living Lab approaches offer new opportunities for the early detection, prevention, and monitoring of these conditions through user-centred innovation and stakeholder collaboration. Our purpose is to review the role of technology in the detection, prevention, and monitoring of geriatric syndromes in older adults with cardiovascular disease and to explore the potential of the Living Lab model for developing and implementing innovative solutions in geriatric care. **Materials and Methods:** A narrative review was conducted using PubMed, CINAHL, MEDLINE, and ScienceDirect. Eleven studies were included. Evidence on physical, cognitive, psycho-emotional, and social frailty, as well as technology-enabled assessment and monitoring approaches, was synthesized. **Results:** Digital technologies, including wearable sensors, telemonitoring platforms, mobile health applications, machine-learning models, and digital phenotyping tools, supported the early identification and monitoring of frailty, fall risk, cognitive decline, depressive symptoms, and functional deterioration. Technology-assisted interventions improved physical and cognitive performance and promoted social engagement. The Living Lab model facilitated the co-creation, evaluation, and validation of technologies in real-world settings, enhancing usability, acceptability, and implementation in clinical practice. **Conclusions:** Technology-supported assessment and monitoring can improve the management of geriatric syndromes in older adults with cardiovascular disease. Living Labs provide a valuable framework for the user-centred development and integration of these innovations, supporting personalized and proactive care strategies that promote healthy ageing.

## 1. Introduction

In the 20th and 21st centuries, medical, pharmaceutical, and technological advances have led to rapid population growth and increased life expectancy, both through reduced infant mortality and increased life expectancy among adults and the elderly [1]. This demographic shift, combined with a reduction in physical activity over the course of a lifetime, makes older adults more vulnerable to diseases and comorbidities associated with a sedentary lifestyle. In this context, frailty has been identified as one of the most serious public health challenges of the century [2].

Frailty has significant consequences for health and the economy. Frail individuals are at increased risk of falls, fractures, reduced mobility, disability, cognitive decline, dementia, depression, hospitalization, institutionalization, and mortality. Furthermore, frailty affects quality of life and is associated with loneliness, highlighting its multidimensional impact beyond clinical and economic consequences [3].

Fried and colleagues defined frailty as “a biological syndrome characterized by a decline in reserves and resistance to stressors, resulting from cumulative declines in multiple physiological systems, leading to vulnerability to adverse outcomes.” Frailty can therefore be understood as the clinical expression of multiple subclinical physiological dysregulations that become evident through impairments in physical performance, as reflected by the five validated frailty criteria. Before the development of overt complications, frailty typically manifests through unintentional weight loss or sarcopenia, muscle weakness, reduced exercise capacity, slowed motor performance, including decreased walking speed, and low levels of physical activity.

Currently, the literature offers numerous definitions of the concept of frailty, ranging from the physical phenotype (weight loss, fatigue, muscle weakness, reduced physical activity, slow walking) to complex approaches that include cognitive function, social conditions, and other aspects. Definitions can be classified into four types: physiological, complex syndromes, balance models (which incorporate social elements), and geriatric syndromes (delirium, falls). Any definition must be multifactorial, dynamic, clinically valid, and computationally tractable; it must correlate with age, sex, comorbidities, and functional status; and it must allow for extrapolation from animal models to human studies. In conclusion, frailty remains a dynamic concept under exploration, and the development of a valid operational definition is essential for research progress and the improvement of geriatric care [4].

Frailty is not merely a deficit but an indicator of adaptability and resilience, underscoring the need for a person-centred approach in geriatric care [5]. Comprehensive geriatric assessment can guide the setting of care goals and the development of a personalized plan, as patients’ needs evolve as frailty progresses, requiring different interventions in diverse contexts. Regular assessment is essential, as frailty is a dynamic process [6,7,8,9]. Frailty is a key vital sign in older patients with cardiovascular disease, but its assessment requires reliable and universally accepted tools to guide clinical decisions. Early detection and intervention regarding frailty, including in the pre-frailty stage, could prevent the progression of cardiovascular diseases and improve patient outcomes [10,11].

This gives rise to the need for an integrative model, with Living Labs being essential for designing solutions tailored to the needs of older adults.

Living Lab approaches have become increasingly relevant in gerontology because they are designed to co-create aging services and technologies with older adults rather than for them. Across the provided literature, Living Labs are positioned as a way to align innovation with real-world needs, improve the relevance of services, and strengthen implementation through iterative feedback and stakeholder collaboration [12,13]. This report addresses the question of what the conceptual foundations and typologies of Living Lab approaches in gerontology reveal about their advantages and limitations, and how these insights can support the development of a theoretical Romanian model for broader ageing services based on a triple helix partnership between an EDIH (European Digital Innovation Hub), a medical university TTO (Technology Transfer Office), and a hospital.

Therefore, this narrative review aimed to examine the role of technology in the detection, prevention, and monitoring of geriatric syndromes among older adults with cardiovascular disease, while exploring the potential of the Living Lab model as a user-centred framework for the development, evaluation, validation, and implementation of these technologies in real-world care settings. Particular attention is given to technological approaches addressing the physical, cognitive, psycho-emotional, and social dimensions of frailty, as well as to the role of Living Labs in facilitating the adoption of innovative solutions tailored to the needs of older adults. Ultimately, these approaches seek to support the early identification of frailty, enable personalized interventions adapted to individual needs, and improve quality of life in the ageing population.

## 2. Materials and Methods

This narrative review was conducted to examine the role of technology and the Living Lab approach in the detection, prevention, and monitoring of geriatric syndromes in older adults with cardiovascular disease.

A literature search was conducted in the PubMed, MEDLINE, CINAHL, and ScienceDirect databases to identify relevant studies published between 2019 and 2025. This time frame was selected to capture recent advances in digital health technologies, including wearable devices, telemonitoring systems, artificial intelligence applications, and Living Lab methodologies, as well as their emerging role in the assessment, prevention, and management of geriatric syndromes in older adults. The search strategy combined terms related to ageing, geriatric syndromes, cardiovascular disease, digital health technologies, and Living Lab methodologies. The search included combinations of keywords such as “older adults”, “elderly”, “ageing”, “frailty”, “geriatric syndromes”, “sarcopenia”, “malnutrition”, “falls”, “cognitive frailty”, “psychological frailty”, “social frailty”, “cardiovascular disease”, “heart failure”, “digital health”, “wearable sensors”, “mobile health”, “telemonitoring”, “artificial intelligence”, “machine learning”, “digital phenotyping”, “Living Lab”, “co-creation”, and “user-centred innovation”. To ensure comprehensive coverage of the literature, additional relevant studies were identified through manual screening of the reference lists of key articles and review papers.

Studies were considered eligible if they addressed the assessment, prevention, monitoring, or management of physical, cognitive, psycho-emotional, or social frailty in adults aged 65 years and older and described the use of digital technologies, sensor-based systems, mobile health applications, artificial intelligence tools, telehealth solutions, or Living Lab methodologies. Original research articles, systematic reviews, narrative reviews, scoping reviews, and relevant reports from international organizations were considered for inclusion.

Publications focusing exclusively on younger populations, non-geriatric conditions, non-health-related technologies, editorials, letters to the editor, conference abstracts without full-text availability, and duplicate records were excluded.

The identified records were screened based on title and abstract relevance, followed by full-text review when necessary. Given the narrative nature of this review, a formal risk-of-bias assessment was not performed. Instead, emphasis was placed on peer-reviewed publications, landmark studies, highly cited articles, and reports that made substantial contributions to understanding the role of technology and Living Labs in the management of geriatric syndromes among older adults.

The literature search initially identified 210 records. After the removal of duplicates and screening for relevance, 140 full-text publications were assessed for eligibility. A total of 11 studies and reports were ultimately included in the narrative synthesis.

## 3. Results

### 3.1. Technology in the Management of Physical Frailty in Older Adults with Cardiovascular Disease

Among all cardiovascular comorbidities, heart failure represents a distinct pathological entity and a geriatric syndrome that, in its progressive stages, is characterized by gradual physical deconditioning. Thus, the progression from early symptomatic stages with the onset of dyspnea, marked fatigue, and later edema, followed by recurrent decompensations and frequent hospitalizations, to the development of malnutrition, depression, cognitive impairment, and functional dependency in advanced stages reflects a continuum of decline from pre-frailty to frailty and ultimately disability [14].

Sarcopenia is a major risk factor for frailty; the two influence each other, sharing common mechanisms such as chronic inflammation, immune dysfunction, oxidative stress, and malnutrition [15]. They are associated with fractures, hospitalizations, disability, and reduced quality of life, with higher risks in vulnerable patients (e.g., those with heart failure) [16]. Physical exercise, especially resistance training, and proper nutrition remain essential interventions.

Regular physical activity and exercise have been shown to have protective effects against multiple components of frailty, improving cognitive function, mobility, sarcopenia, and mood, and sometimes even allowing for the reversal of frailty, both in the community and in nursing homes or during hospitalization. However, more studies are needed to assess the feasibility and effectiveness of these interventions in various clinical populations, especially for frail patients with comorbidities and polypharmacy, who are frequently excluded from studies. In the context of hospitalization, exercise can reduce functional decline and length of stay, but research monitoring the continuity of post-hospitalization interventions is lacking [3].

According to current evidence, pre-frail and frail older adults benefit from multi-component physical activity programs and progressive resistance exercises. Several studies have demonstrated improvements in cognitive function, physical function, and frailty status following exercise-based interventions.

Exergaming and home-based exercise programs have shown favorable results regarding physical and cognitive function. Exercises based on Wii Fit and other interactive platforms outperformed control groups in maintaining or improving physical function.

Regardless of the technological tools employed, physical exercise remains one of the most effective interventions for improving frailty status. Rather than focusing on the development of isolated exercise platforms, digital health innovators should prioritize the integration of these technologies into broader healthcare and coaching ecosystems. Collaboration with healthcare professionals, caregivers, and support services may enhance user engagement, provide personalized guidance, and encourage pre-frail and frail older adults to adopt and maintain higher levels of physical activity, thereby maximizing the long-term benefits of these interventions.

Several studies have used digital tools to facilitate the diagnosis and treatment of physical frailty as technology has advanced and become more accessible [17,18]. Various types of digital sensors for assessing frailty-related parameters are described.

Studies analysing trunk parameters used sensors attached to the chest or lower back to assess sit-to-stand transitions or postural stability. Parameters such as three-dimensional acceleration, angular velocity, and postural balance differed significantly. In their study, Parvaneh et al. used wearable necklace-type sensors placed on the chest of 120 community-dwelling participants over the age of 70 to monitor and assess differences in postural transitions across varying degrees of frailty over a 24 h period. The results showed that the number of stand-to-walk transitions and the total number of postural transitions differed significantly between groups [19].

Gait assessment was another important method for identifying frailty. Parameters such as walking speed, double-support time, step regularity, and maximum speed were associated with frailty status. Thus, Jansen et al. conducted an intervention study in which 112 community-dwelling older adults were asked to wear a sensor integrated into a shirt while performing a gait test under two conditions:

1. Walking a distance of 4.57 m at their own pace.
2. Walking a distance of 10 m as quickly as possible [20]. The results were consistent with previous studies, suggesting that the proportion of time spent walking and standing, the maximum number of steps in a single testing session, and walking speed may represent potential predictors of frailty classification [21].

Other studies on non-gait parameters have assessed upper limb movements and physical activity. Elbow flexion speed, flexibility, muscle strength, and levels of moderate or vigorous physical activity showed significant differences between frail and robust participants. Non-gait parameters demonstrate high clinical feasibility, especially if predictive models could be integrated into a smartwatch or other wearable device.

In their study, Isaradech et al. identified two major categories of digital diagnostic tools: models based on clinical data and models based on biological sensors or wearable devices.

Several studies have explored the use of mobile applications and machine-learning models based on readily available clinical variables, including age, sex, polypharmacy, hospitalization history, and the presence of diabetes mellitus. These approaches demonstrated moderate accuracy in identifying and classifying frailty.

Other studies have employed wearable sensors to assess functional performance, gait characteristics, and balance parameters. The resulting models demonstrated good sensitivity and specificity for the identification of physical frailty, supporting the potential of sensor-based technologies as objective and reliable tools for frailty assessment.

Integrating digital health tools into the diagnosis and management of frailty presents challenges, particularly regarding their adoption by older adults. Another study demonstrated that frailty was associated with both physical activity and the adoption of technology [22]. To counteract frailty, the study suggests that older adults who are less receptive to technology should engage in physical exercise. Another study showed that, although older adults frequently use mobile phones, adoption of wearable devices is low, and 63.2% of the participants assessed were unable to install or delete apps independently. Furthermore, pre-frail and frail older adults use health apps more frequently than healthy individuals of the same age, demonstrating a heightened interest in health-related services and in improving their health and cognitive abilities [23]. These findings highlight the growing interest in developing digital health solutions tailored to the needs of frail older adults. However, for these technologies to be effective in real-world settings, they must be user-friendly, engaging, and accessible. Incorporating elements of gamification and personalized feedback may further enhance motivation, promote sustained participation, and improve long-term adherence among older adults.

Collaborative care models integrated through Atrial Fibrillation-CARE, bringing together geriatrics and cardiology, enable multidimensional assessments, personalized treatment plans, and resource optimization, including non-pharmacological interventions, remote monitoring, and fall prevention strategies, thereby contributing to reduced hospitalizations, prevention of adverse drug reactions, and improved quality of life. In conclusion, current guidelines promote a multidisciplinary, patient-centred approach, emphasizing the need for accurate frailty assessment, adjustment of anticoagulant therapy, and the implementation of collaborative care models to optimize clinical outcomes and address the complex needs of older adults with atrial fibrillation and multiple comorbidities [24].

### 3.2. Technology in the Management of Cognitive Frailty in Older Adults with Cardiovascular Disease

Physical and cognitive frailty interact, influencing the risk of mild neurocognitive disorder, dementia, falls, hospitalization, and mortality.

Thus, common risk factors between cognitive impairment and cardiovascular disease include hypertension, diabetes, obesity, metabolic syndrome, physical inactivity, smoking, as well as psychosocial factors such as depression, social isolation, low educational attainment, and insufficient sleep. These factors simultaneously influence the risk of cardiovascular disease and cognitive decline, creating a bidirectional cycle between physical frailty, cognitive impairment, and cardiovascular disease (CVD). Specific cardiovascular conditions, such as atrial fibrillation and heart failure, increase the risk of cognitive frailty and mild cognitive impairment (MCI), while cardiovascular interventions such as transcatheter aortic valve replacement (TAVR) or valvular surgery may preserve or even improve postoperative cognitive function, particularly when cognitive frailty is assessed prior to the procedure [25,26].

The technological reserve hypothesis posits that engagement with digital technologies promotes better cognitive outcomes than would be expected based on a person’s age, brain injury, or disease stage. There are at least three possible pathways that could link digital engagement to better cognition: complex cognitive stimulation, social connection, and compensatory behaviours. Engagement in cognitively complex activities has long been recognized as associated with better cognitive outcomes [27] as people age, an idea that lies at the heart of reserve theories [28,29]. In 21 articles [30,31,32,33,34,35,36,37,38,39,40,41,42,43,44,45,46,47,48,49] in the literature that compared technology use with other potentially cognitively protective activities, such as reading, games and puzzles, artistic and manual activities, musical engagement, and social activities. In these studies, digital activities were found to have comparable or even stronger associations with positive cognitive outcomes compared to other activities. One possibility is that technological exposures generate more dynamic cognitive stimulation than analog exposures [50,51,52]. For example, both paper crossword puzzles and digital word games involve interacting with cognitively complex information (the puzzle), but digital exposure additionally involves managing constantly changing hardware/software interfaces, resolving device- or internet connection-related issues, and filtering out competing distractions (e.g., text messages or advertisements). Such additional levels of cognitive complexity may explain why older adults who were randomized to learn how to use computers, tablets, social media, or apps (known as digital inclusion interventions) showed significant improvements in episodic memory, processing speed, or overall cognition compared to control groups [53,54,55]. However, not all digital inclusion programs have observed this pattern [56,57,58,59,60], which points to the need for mechanistic studies to better understand the exact processes engaged by different types of technological exposure—an aspect that could inform targeted interventions with rigorously comparable control groups.

A second possible pathway through which technological engagement might protect against cognitive decline is the facilitation of social connection. Better social connection is a well-documented correlate of cognitive functioning in older adults [61].

**Figure 1:**
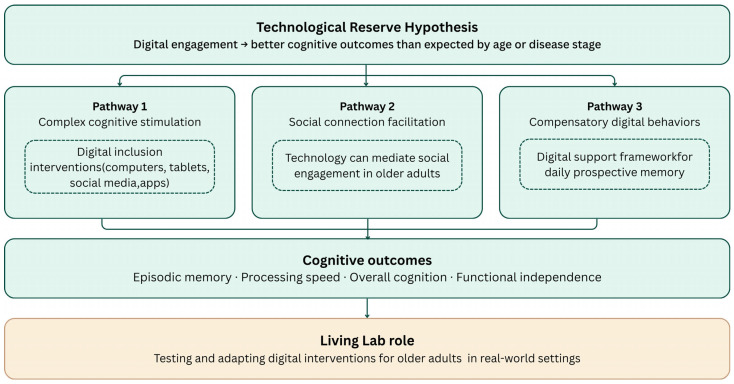
Technology in the Management of Cognitive Frailty.

A third possible pathway is that technology use may promote compensatory behaviors, forming a “digital support framework” [62] that facilitates better functional outcomes in older adults as general cognitive functioning declines. Digital compensatory behaviours are on the rise among older adults [63,64,65] and have been the focus of interventions aimed at supporting daily prospective memory tasks, such as remembering to pay bills on specific dates or taking medications at fixed times each day [66]. Digital support also has implications for understanding clinical diagnoses and disease progression. A testable hypothesis is, therefore, that a technologically enriched environment provides support for individuals with MCI to maintain their independence [67] for longer in certain activities, delaying the diagnostic progression to dementia or reducing the impact of cognitive impairment on daily functioning [68,69]. As clinical practice increasingly embraces the principles of personalized and precision medicine, future research should focus on identifying which patient populations are most likely to benefit from these digital interventions and determining the optimal duration and intensity of support required to achieve meaningful and sustained outcomes [Figure 1].

### 3.3. Technology in the Management of Psycho-Emotional Frailty in Older Adults with Cardiovascular Disease

Although physical and cognitive frailty have been extensively investigated in older adults, these constructs do not fully capture the broader concept of psychological frailty. Psychological frailty arises from age-related declines in both physiological and psychological reserves, leading to increased vulnerability to stressors and a greater susceptibility to adverse health outcomes and disease.

Psychological frailty is a state of mental vulnerability and low resilience resulting from age-related decline in psychological and cognitive reserves. It includes cognitive and emotional factors as well as fatigue, and may coexist with physical, cognitive, or social frailty, but remains a distinct concept. Although interest in this dimension of frailty has grown, there is still no standardized definition or measurement tool [70].

The presence of frailty or pre-frailty is associated with a significantly increased risk of developing long-term depression. This relationship appears to extend beyond the influence of lifestyle and socioeconomic factors, suggesting the involvement of underlying neurobiological and inflammatory pathways that may contribute to the bidirectional interaction between frailty and depressive symptoms.

The relationship between frailty and depression is bidirectional and causal. Frailty increases the risk of depression through effects on mobility, social interaction, and emotional stress, while depression contributes to the onset of frailty by impairing nutrition, sleep, and cognitive health. This complex interaction suggests that simultaneous interventions targeting both conditions may be more effective than treating one of them in isolation [71].

Digital phenotyping can facilitate the early detection of depression and help reduce the high rate of undiagnosed depression (50%) among older adults [72]. Digital phenotyping of mental health is defined as “the moment-by-moment quantification of an individual’s mental health phenotype in real-life contexts using data collected from personal digital devices” [73].

Recent studies have suggested the potential of digital phenotyping for depressive symptoms through the use of momentary ecological assessments, including self-reports of depressive mood [74]. Beyond momentary self-reports, digital detection technologies enable passive and non-intrusive monitoring of depressive symptoms via smartphone apps, wearable sensors, and the Internet of Things. The Internet of Things, a ubiquitous network of interconnected devices, facilitates continuous data collection and intelligent monitoring and management to ensure users’ health and safety [75,76,77,78]. However, previous studies have primarily investigated digital phenotypes of depressive symptoms in young adults or in small groups of patients with depression. This has created difficulties in applying digital phenotyping technologies to older adults [79].

The under-representation of older adults in research on digital mental health is driven by several barriers that limit their participation in studies involving new digital technologies. First, older adults frequently have sensory and cognitive impairments that require the use of design principles different from those effective for young or middle-aged adults [80,81,82]. For example, older adults tend to prefer interfaces that are simple, intuitive, and easy to navigate, with multimodal interaction options, such as voice commands alongside touchscreen controls, to enhance usability and accessibility.

Second, the implementation of digital health services for older adults should involve both family members and community caregivers—who often do not live with the older adult—as well as support from various community health institutions [83,84]. When learning to use new technologies, older adults require repeated face-to-face assistance and educational materials adapted to a low level of digital literacy [80,81]. Digital health services are most likely to benefit older adults when they can connect them with necessary community health services.

Third, the living environment of older adults often hinders their use of digital mental health services, as a significant proportion (40–60%) may lack an internet connection or access to personal computers and other mobile devices [85]. These functional, social, and environmental barriers underscore the need for proof-of-concept and feasibility studies for geriatric mental health services targeting older adults and community caregivers in their natural living environments, using living labs [78,84].

In their study, Song et al. extended beyond feasibility assessment to examine whether digital biomarkers of geriatric depression derived from wearable sensor data could predict day-to-day fluctuations in depressive symptoms among older adults.

### 3.4. Technology in the Management of Social Frailty in Older Adults with Cardiovascular Disease

Social frailty and vulnerability are interconnected, forming a vicious cycle: frail individuals tend to be more socially isolated, with reduced participation in community life and a low perception of social support, and this social vulnerability exacerbates the progression of frailty and the risk of mortality, also affecting cognitive function and emotional well-being. Assessments of frailty must include the individual’s social context, not just physical parameters, as traditional measures do not adequately capture isolation, loneliness, or lack of emotional support; integrating simple social screening tools into clinical practice can enable the early identification of the most vulnerable.

Social frailty and vulnerability are dynamic and can be modified; interventions aimed at either reducing frailty (exercise, nutrition, geriatric management) or reducing social isolation (social prescribing, service navigation) should be evaluated together to understand their mutual impact and optimize long-term clinical and functional outcomes [86].

Recent research (2001–2025) highlights the transition in the study of frailty from analyses of individual variables to multifactorial and integrated models that capture complex interactions between physiological, psychological, behavioral, and social determinants, supporting the use of validated tools such as the Fried Physical Frailty Phenotype and emphasizing the reversibility of early stages through integrated interventions in hospitals, clinics, and communities. Multidimensional models, supported by machine learning technologies and guidelines such as ICOPE, allow for the personalization of frailty assessment and management; but data heterogeneity and limited external validation necessitate continued longitudinal research and testing of combined interventions involving diet, exercise, pharmacotherapy, and psychological support to optimize prevention, reduce progression, and ensure the sustainability of healthcare systems [87].

With regard to social frailty, the literature indicates that specific technological evidence remains relatively limited; however, it identifies several relevant directions for future development. The included studies suggest that digital solutions may support integrated care for older adults through platforms that combine clinical and psychosocial data, thereby facilitating the monitoring of risk factors and the prevention of functional decline. Such platforms have the potential to improve the understanding of the psychosocial dimensions of frailty, including loneliness and social isolation, through the systematic collection and analysis of relevant data [88,89,90].

In addition, the literature highlights that user-centred digital interventions may contribute to reducing feelings of loneliness and social isolation while also supporting mental health and maintaining autonomy. These solutions may facilitate the promotion of active lifestyles and healthy behaviours, as well as enhance autonomy through self-management tools and educational support [88,89,90].

However, the evidence remains primarily conceptual, focusing on the potential of digital platforms rather than on the evaluation of specific interventions targeting social frailty. Therefore, the literature suggests that social frailty remains an underexplored domain from the perspective of dedicated technological solutions, despite increasing recognition of its importance within multidimensional frailty models. This situation underscores the need for empirically validated digital interventions capable of directly addressing social isolation and its determinants in older populations [88,89,90].

### 3.5. The Conceptual Framework of Living Labs for Ageing and Older Adults

**Figure 2:**
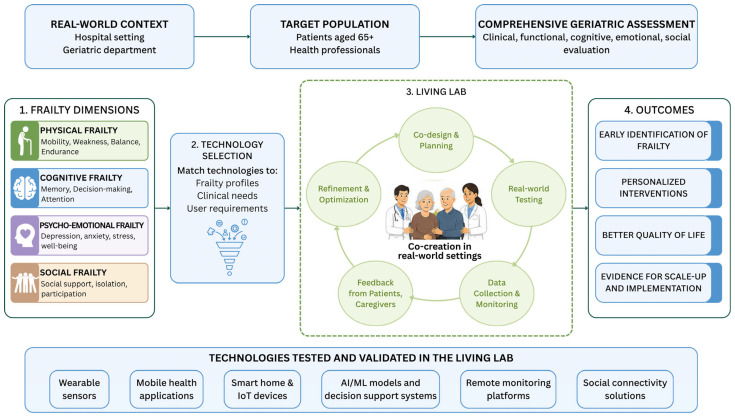
Conceptual framework diagram showing how Living Lab integrates with geriatric syndrome.

The dominant conceptual framing across the literature is that Living Labs are collaborative environments in which older adults, caregivers, researchers, and service providers jointly develop solutions for ageing-related needs [12,91]. The central principles repeatedly emphasized are co-creation, active user involvement, open innovation, and participatory research [12,13]. In this framing, the value of the Living Lab is not simply in testing an intervention in a “real-world” setting, but in making the innovation process itself iterative, relational, and responsive to lived experience [12] [Figure 2].

A key nuance emerging from the literature is that “user involvement” is not treated as a single fixed practice. Instead, older adults may be involved at different levels, from observation and feedback to full participation in design activities [13]. This matters conceptually because the literature treats co-creation as a spectrum rather than a binary condition. The review of methods used in Living Labs with older adults found a broad range of approaches, spanning passive observation through active design participation, and noted that methods may occur over short, mid, or long durations and in temporary or permanent environments such as personal homes, mock-up homes, and community centres [13]. This suggests that the conceptual foundation of Living Labs in gerontology is not tied to one fixed methodology, but rather to a flexible principle of organized participation.

The literature also reveals a subtle tension within the concept itself. On one hand, higher levels of user involvement are valued because they are associated with stronger co-creation. On the other hand, the literature acknowledges that when user needs cannot be readily articulated, less active involvement methods may be necessary and creative thinking may be required [13]. This indicates that Living Labs are not simply user-led systems; they are adaptive systems in which the appropriate mode of participation depends on the clarity of needs, the nature of the innovation, and the stage of the process.

Knight-Davidson et al. provide the methodological foundation for this study by mapping the full spectrum of co-creation methods used with older adults in Living Labs, from passive observation to full participatory design, establishing that inclusive, user-centred approaches combining multiple methods yield the highest co-creation quality, while also highlighting the critical need for methodological adaptability when working with cognitively impaired participants [13].

Figueiredo et al. contribute the only comprehensive structural analysis of digital health Living Labs focused on dementia and cognitive impairment, identifying the tripartite typology (research-driven, testbed, service), documenting the near-total absence of regulatory compliance documentation and sustainability planning across 15 analysed initiatives, and formulating evidence-based operational guidelines directly applicable to the design of AI-integrated gerontological Living Labs [91].

Shin et al. offer the most current and methodologically rigorous integrative review of Living Lab approaches for community-dwelling older adults’ health, synthesising 27 studies against the five canonical LL principles and demonstrating that co-design combined with real-life settings and multi-stakeholder participation constitutes the most effective and feasible configuration for gerontological health innovation [41].

Sengers and Peine contextualise Living Lab development within the broader European innovation ecosystem for age-friendly environments, demonstrating that technological pathways for ageing-in-place are shaped by institutional, regulatory, and market forces, a finding that directly informs the quadruple-helix governance architecture recommended for the SAge Hub Living Lab and its positioning within the Romanian EDIH and medical TTO ecosystem (Table 1) [12].

## 4. Discussion

Across studies, adherence to the five canonical principles—user engagement, multi-stakeholder participation, co-creation, multi-method approaches, and real-life settings—consistently correlates with higher relevance and adoption of produced solutions. The integrative review by Shin et al. confirmed that full tripartite co-creation, engaging older adults, researchers, and external stakeholders simultaneously, was achieved in the majority (16/27) of reviewed studies, signalling a maturation of the methodology beyond its earlier user-centred design roots [41]. Similarly, Knight-Davidson et al. demonstrated that the breadth of methods deployed in gerontological LLs, from ethnographic shadowing and World Café facilitation to ICT-mediated scenario testing and participatory diaries, reflects a recognition that older adults are not a homogeneous group and that methodological flexibility, rather than standardisation, is the operational hallmark of effective co-creation [13].

However, convergence on principles does not equate to methodological consensus. A critical observation across all four reviewed sources is that terminological fragmentation remains a fundamental epistemological barrier: co-creation, co-design, co-production, participatory action research, and user-centred design are employed interchangeably across the literature, yet they embed substantively different philosophical commitments regarding the locus of power in innovation. This semantic inconsistency impedes cross-study comparability, complicates systematic review methodology, and may distort policy uptake of LL evidence. Furthermore, only one of the 27 studies in Shin et al. explicitly used the term “living lab,” despite the majority satisfying its definitional criteria. This semantic gap between practice and label suggests that the published evidence base substantially under-represents the real volume of LL-aligned gerontological research globally, a limitation with direct implications for the apparent nascency of the field [41].

A second critical finding concerns co-creation depth. Knight-Davidson et al. [13] propose a co-creation continuum ranging from passive user observation at one end to full ownership of design challenges at the other. The evidence suggests that most gerontological LLs operate in the mid-range of this continuum—involving older adults as active collaborators but rarely positioning them as initiators of innovation problems. This is not merely a methodological shortfall; it reflects a structural asymmetry between the institutional resources of researchers and the participatory capital of older adults, particularly those with cognitive impairment or limited digital literacy [13]. Figueiredo et al. observed that none of the 15 analysed digital health LLs measured whether participants felt like equal partners in the process, and that in all dementia-focused studies, the initial technological concept had been preconceived before user inclusion [91]. This represents a significant departure from the emancipatory ideal embedded in the LL paradigm and points to an unresolved tension between research efficiency and genuine co-determination.

Four key structural gaps warrant explicit consideration. First, regulatory and ethical governance remains insufficiently developed. Across 15 digital health LLs, Figueiredo et al. [91] reported that none explicitly documented comprehensive data privacy and security measures, while only two addressed compliance with relevant regulatory frameworks. In the context of the EU AI Act and GDPR obligations, this constitutes not merely a gap but a compliance risk that threatens the long-term legitimacy of LL research, particularly when vulnerable populations are involved. Second, sustainability planning is almost universally absent: only one of the 15 analysed LLs (Laval-ROSA Transilab) had documented post-project sustainability strategies, confirming that LL lifecycles remain overwhelmingly coterminous with their funding cycles—a configuration that systematically erodes accumulated relational and methodological capital. Third, standardised outcome metrics are lacking: while most LLs report impact narratives, fewer than half maintain clear, reproducible success metrics aligned to healthcare outcome frameworks. This absence renders cross-site learning and meta-analytical synthesis methodologically precarious. Fourth, the geographic and socioeconomic distribution of LL evidence is highly skewed: the overwhelming majority of high-quality studies originate from Western and Northern Europe, Australia, and North America, while evidence from Central and Eastern European contexts—including resource-constrained public health systems, low digital penetration among older cohorts, and fragmented institutional landscapes—remains virtually absent from the peer-reviewed literature [91].

The literature does not converge on a single, universally accepted typology of Living Labs in gerontology; rather, multiple overlapping classification frameworks coexist. Figueiredo et al. propose a structural tripartite typology (research-driven, living testbed, and Living Lab as a service), finding that among 15 dementia-focused digital health LLs, 12 were research-driven, 9 qualified as testbeds, and 3 as service LLs, with several fitting multiple categories simultaneously [91]. Complementarily, Schuurman et al. (as cited in Knight-Davidson et al.) offer a quadruple typology based on organisational and user-involvement logic: American LLs, testbed-like LLs, co-creation–intensive LLs, and facilitator/knowledge-sharing LLs [13]. At the methodological level, Shin et al. and Knight-Davidson et al. propose a continuum typology based on the degree of user involvement—from user-centred/expert-driven at one pole, through participatory design and co-design, to full co-creation and co-production at the other—noting that most gerontological LLs cluster in the co-design range, with only a minority achieving genuine co-determination of research agendas [13,41]. The Maastricht Living Lab on Ageing and Long-Term Care illustrates a further institutional typology: the academic-practice translational LL, structured around Linking Pin roles and team science. These classifications are not mutually exclusive; a single LL may simultaneously be research-driven (Figueiredo), co-creation–intensive (Schuurman), and translational (Maastricht), depending on the analytical lens applied.

The Maastricht Living Lab in Ageing and Long-Term Care is a reference point because it shows how Living Lab principles can be translated into a durable institutional structure. The Maastricht model is presented as a sustainable interdisciplinary collaboration between scientists, care providers, and educators in long-term care, with the explicit mission of improving quality of life, quality of care, and quality of work [95]. Its key mechanisms are the Linking Pins and an interdisciplinary team-science approach, which allow research and practice to remain continuously connected [95]. Importantly, the model is not a short-term pilot but a long-standing infrastructure that has operated for more than 25 years and has been replicated in other countries [95].

This institutional durability is precisely why Maastricht is more than a descriptive case study. It demonstrates that a Living Lab in ageing succeeds when collaboration is formalized, roles are clearly distributed, and the model is embedded in everyday care practice [95]. The Maastricht example also shows that sustainability is not a secondary issue; it is part of the model’s core architecture [95]. For the present paper, this is especially relevant because the Romanian Living Lab requires an implementation model that can survive beyond a single project cycle and can support both innovation and clinical translation (Table 2).

The Maastricht Living Lab model in ageing shows that a robust Living Lab in elderly care requires a formal partnership between universities, long-term care providers, schools of health professions, and community stakeholders, with clear mechanisms for governance, data sharing, and prioritization of the research agenda. Contemporary European initiatives, such as the SAge-Hub program for smart ageing [96], show that defining a portfolio of regionalized Living Labs (e.g., 4 Living Labs for health and smart ageing) is linked to empowering healthtech SMEs and public actors through training and innovation support.

The literature on Living Labs as tools for social innovation shows that older adults’ involvement is not limited to testing, but includes co-identifying problems, co-designing services, and participating in impact assessment, which strengthens the relevance and acceptability of interventions. In the context of ageing, these participatory processes are important for addressing not only medical issues but also social ones, such as loneliness, declining community participation, and the need for emotional support, which have been highlighted as risk factors in studies on the psychosocial health of older adults.

Reports and guidelines on the implementation of Living Labs in healthcare [97] highlight the need for data protection frameworks, informed consent tailored to older adults, transparency regarding data use, and the involvement of patient organizations and families in project governance. Furthermore, the literature on innovation in long-term care highlights the importance of balancing continuous monitoring (via sensors) with respect for the older adult’s autonomy and privacy, particularly in residential settings or at home.

From the perspective of the EU AI Act, such a system, used for diagnosis, risk stratification, and prioritization of clinical interventions, falls into the category of high-risk systems, alongside other clinical decision-making and medical monitoring applications. This classification entails strict requirements regarding risk management, data governance, transparency, robustness, human oversight, and post-market documentation.

The indicators used will be anchored to a comparative clinical standard. The gold standard includes functional and balance tests, frailty scores, cognitive assessments, nutritional parameters, and mental health indicators [98] (comprehensive geriatric assessment), and the data generated by the AI system and sensors are systematically compared with these benchmarks under real-world conditions. Complementarily, psychosocial indicators (social participation, self-perceived health, depressive symptoms) are integrated, given the documented association between depression, functional dependence, history of falls, malnutrition, and frailty in older adults. In line with the EU AI Act [99], these components contribute to assessing the impact on fundamental rights and to the early identification of potential adverse effects that extend beyond the strictly clinical domain (e.g., loss of autonomy or digital exclusion), an aspect relevant to the obligation to assess and monitor risks to health and rights.

From an EU AI Act compliance perspective, the Living Lab framework provides the empirical conditions required to validate high-risk AI system requirements—including human oversight effectiveness, alert proportionality, data governance robustness, and the transparency of AI-generated recommendations for both clinicians and patients—under realistic, ecologically valid care conditions.

The literature suggests that validation in a geriatric Living Lab must be broader than algorithmic performance. In dementia-focused Living Labs, multiple stakeholders are involved, including academic institutions, private companies, health care organizations, and patient representative bodies, and the main purpose is to support health, independent living, safety, and caregiver burden reduction [100]. However, this literature also shows that the quality of interventional evidence remains limited, and that more rigorous studies are needed to demonstrate effectiveness [100]. This is relevant to the present work, because a Living Lab for frailty and other geriatric syndromes should validate both clinical outputs and implementation outcomes.

Accordingly, validation should include clinical agreement between the digital system and standard geriatric assessment, as well as measures of usability, acceptability, adherence, and integration into workflow. The need for impact measurement is reinforced by a recent review of digital health-focused Living Labs, which identifies user engagement, interdisciplinary collaboration, technological infrastructure, regulatory compliance, transparent innovation processes, impact measurement, sustainability, scalability, dissemination, and financial management as core requirements for effective Living Labs [91]. Programs such as SAge-Hub [96] emphasize the generation of “evidence and performance data” in the Living Lab, usable by investors and institutional clients, which involves standardized evaluation protocols and transparent reporting.

We can thus argue that early validation in the Living Lab is not merely a “nice-to-have,” but a structural component of the responsible development of high-risk health systems (according to the EU AI Act), providing an environment where uncertainties regarding performance, usability, and ethical impact can be explored in a controlled manner, with the participation of all relevant stakeholders.

Living labs are essential for designing solutions tailored to the needs of older adults. Digital mental health services will not be acceptable or sustainable unless they are designed to be compatible with the cognitive and physical capabilities of older adults, their natural living environments, and the work environments of community caregivers. This compatibility can be ensured through testing in living labs [100,101].

## 5. Future Directions

Drawing on the typological evidence synthesized above, the most methodologically coherent approach for a Living Lab in the Smart Ageing sector—such as the one envisioned within the SAge Hub project—is a co-creation-intensive, academic practice translational model, anchored in a real-world clinical infrastructure. The literature consistently demonstrates that Living Labs achieving the highest ecological validity and regulatory robustness are those that embed innovation directly within operational care environments rather than parallel or simulated ones. Accordingly, the recommended configuration involves a structured partnership between a university medical faculty (Gr.T. Popa University of Medicine and Pharmacy, Iași), a clinical host institution (Parhon Hospital, cardiology, geriatrics, and outpatient departments), a Technology Transfer Office (MAVIS TTO), and a European Digital Innovation Hub (EDIH—Digital Innovation Zone)—a quadruple-helix architecture empirically validated as the governance baseline for sustainable gerontological LLs. The innovation object is a clinical decision support system based on artificial intelligence, designed for older patients (≥65 years) with cardiovascular disease, integrating clinical, functional, and digital data for the early identification of frailty risk, falls, cognitive decline, and nutritional deterioration. In alignment with the translational Linking Pin model [95] documented by the Maastricht Living Lab on Ageing and Long-Term Care, the recommended approach positions clinical professionals as co-investigators rather than passive adopters, ensuring that the AI system is iteratively refined through continuous bidirectional feedback between research and practice. Consistent with Shin et al.’s (2021) finding that the most effective gerontological LLs combine co-design with real-life settings and multi-method evaluation, validation should extend beyond algorithmic performance to encompass AI–clinician agreement, risk detection sensitivity, time to intervention, changes in functional scores, device wear adherence, data completeness, and acceptability—assessed under real-world care conditions with longitudinal follow-up that captures variation in behaviour, comorbidities, and social context. This multi-level outcome architecture responds directly to what Figueiredo et al. identify as the dominant gap across reviewed dementia and ageing-focused LLs: the absence of clear success metrics and evidence of achieved outcomes, documented in fewer than half of analysed initiatives [91]. Furthermore, given that none of the 15 LLs reviewed by Figueiredo et al. documented robust data privacy and security measures, and in light of the EU AI Act’s requirements for high-risk system performance and robustness demonstration, the recommended approach explicitly integrates regulatory compliance—GDPR data governance, ethical review, and AI Act conformity assessment—as a structural component of the LL design rather than a post hoc consideration, in line with the approach advocated by Knight-Davidson et al. for authentic, principle-adherent Living Lab operation [13].

Several implementation barriers specific to the Romanian context must be foregrounded. Institutional trust deficits represent perhaps the most fundamental: the Maastricht model explicitly identifies reciprocity and trust among partners as the driving force of LL sustainability, and cautions that major results should not be expected in the early years. In a Romanian context where inter-institutional collaboration has frequently been disrupted by staff turnover, grant cycle discontinuities, and political volatility in health administration, trust-building cannot be assumed—it must be designed, through formalised governance structures, contractual role definitions, and managed expectations from Year 1. Digital exclusion of older adults constitutes a second structural barrier: adoption of new technologies by older Romanians remains a goal rather than a reality, necessitating an analogue-first, technology-second methodological stance that most Western LL models do not prioritise. Power imbalances in co-creation partnerships are particularly acute when participants are older adults from a healthcare system culturally oriented toward paternalistic patient relationships; Shin et al. identify this as a recurrent barrier and recommend explicit role-assignment mechanisms and ongoing consent procedures calibrated to participatory dynamics [41]. Finally, sustainability beyond project funding—the most pervasive failure mode across all reviewed LLs—is structurally amplified in Romania by the dependency of research infrastructure on EU funding cycles, underscoring the imperative of integrating CNAS and Ministry of Health as co-investors in LL infrastructure from the earliest phases of development.

## 6. Limitations

Several limitations of this review should be acknowledged. First, this work was conducted as a narrative review rather than a systematic review. Although a structured literature search was performed, the processes of study selection and evidence synthesis remain inherently vulnerable to selection bias. In addition, no formal risk-of-bias assessment was undertaken. Consequently, the findings should be interpreted as a qualitative synthesis of the current literature, intended to provide a broad overview of the field rather than a quantitative appraisal of the available evidence.

Second, the studies included in this review were highly heterogeneous in terms of study design, participant characteristics, clinical settings, technologies evaluated, and outcome measures. The available evidence covered a broad spectrum of digital health interventions, including wearable sensors, telemonitoring systems, mobile health applications, artificial intelligence tools, digital phenotyping approaches, and Living Lab initiatives. This heterogeneity limits direct comparisons across studies and prevents definitive conclusions regarding the superiority of specific technologies or implementation models.

Third, although the Living Lab concept is increasingly recognized as a promising framework for the co-creation and validation of health innovations, evidence specifically addressing its application to individual geriatric syndromes remains limited. In several areas, particularly psycho-emotional and social frailty, the available literature is still relatively sparse, with most studies focusing on feasibility, usability, acceptability, and implementation processes rather than on long-term clinical effectiveness and patient outcomes.

Another limitation relates to the contextual nature of the proposed Living Lab model. The discussion and implementation framework presented in this review are largely informed by European experiences and by the characteristics of the Romanian healthcare and innovation ecosystem. Consequently, differences in healthcare organization, digital infrastructure, regulatory frameworks, and resource availability may affect the generalizability and transferability of these findings to other countries and healthcare settings.

Finally, the field of digital health is evolving rapidly. Technological innovations, advances in artificial intelligence, changes in regulatory frameworks, and the continuous development of new monitoring devices may lead to the rapid obsolescence of some of the evidence reviewed. Consequently, the conclusions of this review should be interpreted within the context of the currently available literature and may require periodic updating as new evidence emerges.

Technology use in older adults with cardiovascular disease remains an underexplored area in the current literature, despite its growing relevance for contemporary clinical practice. It represents a significant gap, particularly in relation to the individualization of therapeutic strategies and the optimization of patient-centred care. Addressing this gap is essential for informing future research and for the development of tailored, evidence-based interventions in this high-risk population.

## 7. Conclusions

By integrating physical, cognitive, psychological, and social health data, Living Labs for older adults can support a comprehensive approach to the management of geriatric syndromes. Within this framework, risks can be identified at an early stage, while interventions are co-designed, evaluated, and continuously refined through feedback generated in real-world settings, ensuring that solutions remain responsive to the evolving needs of older adults.

For older adults with cardiovascular disease, in whom frailty is associated with recurrent hospitalizations, functional decline, disability, and reduced quality of life, such a multidimensional and integrated assessment framework is likely to be more meaningful than reliance on a single algorithmic metric.

## Figures and Tables

**Table 1:** Synthesis of the Main Studies Analysed for Each Domain of Frailty.

Frailty Domain	Author (Year)	Study Type	No. Participants	Technology Used	Main Finding
*Cognitive frailty*	Benge & Scullin (2025)	Meta-analysis	411,430 (across 57 meta-analysed studies);	Digital technologies including computers, internet, smartphones, and social media platforms [65,66].	Greater use of everyday digital technologies was associated with reduced odds for cognitive decline, operationalized as lower cognitive test scores and reduced mild cognitive impairment or dementia diagnoses [65,66].
*Cognitive frailty*	Jin et al. (2019)	Longitudinal observational study	13,457	Desktop computer with internet connection at home; mobile/cellphone [92].	Findings from this study underscored the importance of digital devices as a platform for cognitively stimulating activities to delay cognitive decline [92].
*Cognitive frailty*	Pressler et al. (2019)	Narrative review	Not applicable—design study	BrainHQ program, developed by PositScience, delivered by computer, home-based [93].	Preliminary studies using BrainHQ demonstrated improvements in memory and working memory, and increased serum BDNF levels over 12 weeks in two small samples of patients diagnosed with heart failure. Viewing nature images on a laptop improved attention in both heart failure patients and healthy control participants [93].
*Cognitive frailty*	Figueiredo et al. (2024)	Narrative review	Not applicable—design study	Assistive technologies, remote monitoring systems, smart home appliances, health monitoring apps, wearables, sensors, robots, digital diagnostics, digital therapeutics [91].	15 digital health Living Labs focused on dementia were identified and analysed; key challenges include limited scalability, lack of systematic evaluation, and poor healthcare system integration; guidelines were proposed emphasizing user-centric co-creation, interdisciplinary collaboration, regulatory compliance, transparent innovation, sustainability planning, and financial management to enhance Living Lab effectiveness in dementia care [91].
*Cognitive frailty*	Knight-Davidson et al. (2020)	Scoping review	Not applicable—design study	ICT (mobile apps, smartphones, tablets), assistive living technologies telehealth/personal health systems, wearable alarms, sensor technology, VR/3D simulation, smart TV platforms [13].	Inclusive, user-centred methods with high active user involvement are most effective for needs-finding and co-creation with older adults; a flexible repertoire of methods is recommended, as no single approach suits all contexts, particularly given heterogeneity among older adults (e.g., cognitive decline, trust barriers) [13].
*Physical frailty*	Isaradech & Sirikul (2025)	Narrative review	Individual studies range from N = 23 to N = 718	Combination of wearable inertial sensors, Android mobile applications, machine learning models, active gaming platforms for rehabilitation, home telemonitoring systems [94].	Digital health tools improve early frailty detection through sensor-derived physical metrics, while exergaming and home-based programs significantly enhance physical and cognitive functions. Although technology acceptance remains a challenge for older adults, future success depends on developing user-friendly, integrated platforms that combine frailty screening with personalized preventive care [94].
*Physical frailty*	Shin et al. (2021)	Integrative review	Not applicable—design study	mHealth, eHealth self-management platforms, (SMS) message banks, ICT robotics, Embodied Conversational Agents, web-based exercise programs, digital talking books, cloud servers and smart assistive mobility devices [41].	Living labs demonstrated feasibility and effectiveness in geriatric community care; co-creation methodologies were successfully applied across 27 studies to develop health solutions for older adults, with most adhering to the five living lab principles [41].
*Social frailty*	Kouroubali et al. (2022)	Methodological Study	Not applicable—design study	An AI-enabled integrated platform (Elder Care Platform) incorporating mobile and web applications, smartwatch and biometric sensors, electronic health records (EHR), machine learning-based frailty risk models [90].	Digital solutions support integrated care, early frailty detection, and the prevention of disability and hospital admissions. Platforms must be holistic, addressing both physical condition and psychosocial factors like loneliness and isolation. User-centred, tailored digital interventions enhance self-management, education, and user empowerment. Collected data can be anonymized and used for research to create an evidence-based roadmap for frailty [90].
*Social frailty*	Sato et al. (2022)	Retrospective cohort study	26,357	Kihon Checklist (KCL)—25-item self-administered questionnaire [89].	The Kihon Checklist, particularly its IADL, locomotor, and cognitive function domains, significantly predicts transition to severe long-term care dependency among community-dwelling older adults, supporting its use as a practical screening tool for early identification of high-risk individuals in community health settings [89].
*Social frailty*	Bessa et al. (2021)	Longitudinal prospective cohort study	180	Not specified in article [88].	Social criteria used in the study act as both predictors and criteria of frailty. Lack of perceived social support, loneliness, and decreased social activities longitudinally predict frailty. Lack of social relations and living alone did not predict frailty; their inclusion requires further research [88].
*Social frailty*	Sengers & Peine (2021)	Embedded multiple-case study	Not applicable—design study	Smart home devices, sensors, fall detectors, telecare systems, interactive ICT devices, robots, smart rollators, smart beds [12].	Most innovations tested across 53 European age-friendly home experiments are primarily social or conceptual rather than technological in character; seven innovation pathways were identified, with social innovations (e.g., intergenerational co-housing, home sharing, community-based models) appearing more transformative than technology-focused approaches for enabling older adults to age in place independently [12].

**Table 2:** Comparison of the Cited Living Lab Models.

Dimension	Main Pattern in the Literature	Implication for a Future Romanian Adapted Model
Conceptual basis	Living Labs are defined by co-creation, user involvement, open innovation, and participatory research, and older adults may be involved across a continuum from observation and feedback to active design participation (Sengers & Peine, 2021) (Figueiredo et al., 2024) (Knight-Davidson et al., 2020) [12,13,91].	Living Labs should be framed as a participatory process, not merely as a physical setting; the model must allow staged, iterative engagement rather than assuming one fixed form of participation.
Typology and governance	Living Lab configurations vary by setting, governance, stakeholder composition, intervention stage, and participation intensity (Knight-Davidson et al., 2020) (Sengers & Peine, 2021) [12,13]. The Maastricht Living Lab in Ageing and Long-Term Care is a long-term translational model built on interdisciplinary collaboration, linking pins, and embedded research-practice integration (Verbeek et al., 2020) [95].	The Romanian model should be framed as a locally adapted governance architecture rather than a direct transfer of Maastricht; Maastricht is best used as a benchmark for institutional continuity, role clarity, and translational embedding.
Expected advantages	The strongest gerontological Living Labs combine co-design, real-life settings, and multi-stakeholder participation (Sengers & Peine, 2021) [12]. Across the literature, active and iterative participation is associated with greater relevance, acceptability, feasibility, and empowerment (Knight-Davidson et al., 2020) [13].	The principal value of the model lies in improved fit between innovation and lived need; in gerontology, this is especially important because frailty, multimorbidity, and variable digital literacy make standardized top-down solutions less effective.
Limitations and risks	Reported limitations include recruitment bias, representativeness problems, sustainability challenges, power imbalance, resource intensity, evaluation difficulty, and transferability constraints (Knight-Davidson et al., 2020) [13]. Co-research with older adults is also methodologically demanding and labour-intensive (James & Buffel, 2022), (Sengers & Peine, 2021) [11,12].	Credible implementation requires methodological and governance safeguards: transparent recruitment, balanced participation, explicit role definition, and predefined evaluation criteria that can support transfer beyond a single pilot site.
Operational priorities	Digital health-focused Living Labs require user engagement, interdisciplinary collaboration, infrastructure, compliance, impact measurement, dissemination, and financial sustainability ((Figueiredo et al., 2024), (Shin et al. 2021)) further show that the most effective configuration for older adults’ health is co-design embedded in real-life settings with multi-stakeholder participation [41,91].	Scaling depends on system-level support, not on technology alone; the Romanian partnership should therefore function as an implementation system with governance, compliance, adoption support, and sustainability planning built in from the outset.

## Data Availability

No new data were created or analysed in this study. Data sharing is not applicable to this article.
